# S3 Guideline for the Treatment of Psoriasis vulgaris, adapted from EuroGuiDerm – part 2: Specific clinical and comorbid situations

**DOI:** 10.1111/ddg.16001

**Published:** 2026-02-05

**Authors:** Alexander Nast, Andreas Altenburg, Matthias Augustin, Frank Bachmann, Wolf‐Henning Boehncke, Markus Cornberg, Hilte Geerdes‐Fenge, Brit Häcker, Peter Härle, Joachim Klaus, Michaela Köhm, Arno Köllner, Ulrich Mrowietz, Hans‐Michael Ockenfels, Antonia Pennitz, Sandra Philipp, Thomas Richter, Thomas Rosenbach, Tom Schaberg, Martin Schlaeger, Gerhard Schmid‐Ott, Michael Sebastian, Karisa Thölken, Ralph von Kiedrowski, Uwe Willuhn, Christoph Zeyen

**Affiliations:** ^1^ Division of Evidence‐Based Medicine (dEBM) Department of Dermatology Venereology and Allergology Charité – Universitätsmedizin Berlin corporate member of Freie Universität Berlin and Humboldt‐Universität zu Berlin Berlin Germany; ^2^ Department of Dermatology Venereology and Allergology Dessau Municipal Hospital Dessau Germany; ^3^ Institute for Health Services Research in Dermatology and Nursing (IVDP) University Medical Center Hamburg‐Eppendorf (UKE) Hamburg Germany; ^4^ Dermatology Center Berlin Siddi & Bachmann Berlin Germany; ^5^ Service de Dermatologie et Vénéréologie Hôpitaux Universitaires de Genève Geneva Switzerland; ^6^ Department of Gastroenterology Hepatology and Endocrinology Hannover Medical School Hannover Germany; ^7^ Department of Internal Medicine, Tropical Medicine and Infectious Diseases Rostock University Medical Center Rostock Germany; ^8^ German Central Committee against Tuberculosis (DZK) Berlin Germany; ^9^ Rheumatology Center according to G‐BA Department of Rheumatology Clinical Immunology and Physical Therapy Marienhaus Hospital Mainz Mainz Germany; ^10^ German Psoriasis Federation Hamburg Germany; ^11^ Division of Rheumatology Immunology – Inflammation Medicine University Hospital Frankfurt Goethe University Frankfurt am Main Germany; ^12^ Fraunhofer Institute for Translational Medicine and Pharmacology ITMP Frankfurt Frankfurt am Main Germany; ^13^ Dermatology Office Duisburg Germany; ^14^ Psoriasis Center Department of Dermatology Venereology Allergology University Hospital Schleswig‐Holstein Campus Kiel Kiel Germany; ^15^ Department of Dermatology and Allergy Hospital Hanau Hanau Germany; ^16^ Dermatology Office Oranienburg Germany; ^17^ Helios Versorgungszentren GmbH MVZ Gotha Gotha Germany; ^18^ Dermatology Office Osnabrück Germany; ^19^ Dermatology Office Oldenburg Germany; ^20^ Berolina Hospital Löhne Germany; ^21^ Dermatology Office Mahlow Germany; ^22^ Department of Dermatology and Allergology University Hospital Augsburg Augsburg Germany; ^23^ Dermatology Office Selters Germany

**Keywords:** Comorbidity, hepatitis, psoriasis, recommendations, tuberculosis

## Abstract

The present Part 2 of the updated German S3 guideline on the treatment of psoriasis vulgaris provides recommendations for therapy selection in special clinical situations and in the presence of comorbidities. A major focus of this update is the chapter on screening for tuberculosis as well as therapy selection and management in latent tuberculosis. The recommendations regarding the use of interferon‐gamma release assays and the indication for chest radiography have been extensively revised. In addition, the guidance on the suitability of systemic psoriasis therapies in patients with latent tuberculosis and on the need for preventive antituberculous treatment has been thoroughly updated. In the chapter on inflammatory bowel diseases, risankizumab and guselkumab have been added as recommended treatment options, as both agents have recently been approved for the indications Crohn's disease and ulcerative colitis. Further substantial revisions are included in the chapters on patients with a history of malignancy and viral hepatitis.

## NOTES ON USING THE GUIDELINE

This publication includes selected chapters and text passages where particularly relevant modifications have been made. Apart from the sections in part 2 presented here, part 1 includes the chapters “Disease severity and treatment goals”, “Initiating and selecting a systemic therapy”, “Overview of treatment options”, “Results of the network meta‐analysis”, and the “Instructions for use of the individual drugs”.

The long version of the guideline is available on the AWMF pages (https://register.awmf.org/de/leitlinien/detail/013‐001). Particular attention concerning the use of the guideline recommendations presented in this short version should be paid to the information provided in the chapter “Notes on using the guideline/Disclaimer” of the long version. The following accompanying documents of version 8 are available on the AWMF pages: Appendix A (“Recommendations for topical therapy”, “Phototherapy”, “Other therapies”, “Interface definition”), Evidence Report, Guideline Development Report with information about conflicts of interest, set of PowerPoint slides on guideline implementation.

*For the section “Notes on using the guideline/Disclaimer” (these apply equally to the present short versions), see the long version*.

## SPECIFIC CLINICAL AND COMORBID SITUATIONS

Tables 1 and 2 provide an overview of the selected specific clinical decision‐making situations and the assessment of the guideline group concerning these medications. The colors and symbols used in Tables 1 and 2 are explained in Table 3.

**TABLE 1:** Decision grid (I) on conventional therapies with expert consensus on their suitability in special clinical situations.

	Conventional systemic therapeutics			
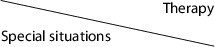	**Acitretin**	**Ciclosporin**	**Fumarates**	**Methotrexate**
**Chronic inflammatory bowel disease: Crohn's disease**	**↑** especially cases with mild paradoxical psoriasis			**↑**
**Chronic inflammatory bowel disease: ulcerative colitis**	**↑** especially cases with mild paradoxical psoriasis	**↑**		
**Diabetes mellitus /metabolic syndrome**		Consider alternatives		Consider alternatives
**Dyslipidemia**	**↓**	**↓**		
**Advanced heart failure**	**↑**	**↓**		**↑**
**Heart disease: ischemic heart disease**	**↓**	**↓**		**↑**
**Latent TB infection / treated TB**	**↑↑**	**↑↑**	**↑↑**	**↑↑**
**Pregnancy**	**↓↓**		**↓**	**↓↓**

**TABLE 2:** Decision grid (II) on biologics and new targeted small molecules with expert consensus on their suitability in special clinical situations.

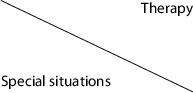	PDE‐4 inhibitor	TYK‐2 inhibitor	TNF inhibitors				IL12/23p40 inhibitors	IL17 inhibitors				IL23 inhibitors		
	Apremilast	Deucravacitinib	Etanercept	Infliximab	Adalimumab	Certolizumab	Ustekinumab	Secukinumab	Ixekizumab	Brodalumab	Bimekizumab	Guselkumab	Tildrakizumab	Risankizumab
**Chronic inflammatory bowel disease: Crohn's disease**				**↑↑**				**↓**				**↑↑**	**↑**	**↑↑**
**Chronic inflammatory bowel disease**: **ulcerative colitis**	**↑**			**↑↑**			**↑↑**	**↓**				**↑↑**	**↑**	**↑↑**
**Diabetes mellitus / metabolic syndrome**														
**Dyslipidemia**														
**Advanced heart failure**	**↑**		**↓↓**				**↑**							
**Heart disease: ischemic heart disease**			**↑**											
**Latent TB infection / treated TB**	**↑↑**	**↓**	**↓↓**				**↑**							
**Pregnancy**	**↓**	**↓**				**↑**								

**TABLE 3:** Legend for Table 1 and Table 2.

Symbols	Implication
**↑↑**	We believe that all or almost all informed people would make a choice in favor of using this intervention. Clinicians will not have to spend as much time on the process of decision‐making with the patients. In most clinical situations, the recommendation can be adopted as a policy.
**↑**	We believe that most informed people would make this decision, but a substantial part would not. Clinicians and other health care providers will need to devote more time to ensure that the choice of the procedure together with the potential consequences reflects the values and preferences of individual patients. Decision processes in the health system require deeper discussions and involvement of stakeholders.
	See background text and specific recommendations in the respective chapters.
**↓**	We believe that most informed people would make a choice against using this intervention, but a substantial number would not.
**↓↓**	We believe that all or almost all informed people would make a choice against using this intervention. In most clinical situations, the recommendation can be adopted as a policy.

### Overwiew of the Topics and Questions to be Addressed in Comorbid and Special Clinical Situations

**Table:** 

**TOPIC**	**QUESTIONS**
Chronic inflammatory bowel diseases	How should psoriasis patients with concomitant inflammatory bowel disease be managed?
Cancer	How should psoriasis patients with a history of malignancies be managed?
Depression	How should psoriasis patients with depression and/or suicidal ideation be managed?
Diabetes mellitus	How should psoriasis patients with diabetes mellitus be managed?
Heart disease	How should psoriasis patients with CHD and/or heart failure with reduced ejection fraction (HFrEF) be managed?
Kidney disease	How should psoriasis patients with kidney failure/renal impairment be managed?
Neurological diseases	Which treatments are appropriate for psoriasis patients with neurological diseases?
Viral hepatitis	When and how should psoriasis patients be screened for viral hepatitis and how should patients who test positive be managed?
Tuberculosis screening	How to screen for tuberculosis before and during systemic therapy?
Tuberculosis and therapy	How should psoriasis patients with positive interferon‐gamma release assay (IGRA) be managed?
Wish for child/pregnancy	How should psoriasis patients with a wish for pregnancy in the near future or who are pregnant be managed?
Vaccinations	How should vaccinations be managed in psoriasis patients on systemic treatment?

## INFLAMMATORY BOWEL DISEASE: HOW SHOULD PSORIASIS PATIENTS WITH CONCOMITANT INFLAMMATORY BOWEL DISEASE BE MANAGED?

After open consultation, the agents risankizumab and guselkumab were reassessed based on the approval status of psoriasis therapies for Crohn's disease and ulcerative colitis. For the explanatory background text to this chapter, see the long version.

**Recommendations**:

**Table:** 

3.1‐1 | reviewed [2025] *We recommend* working in collaboration with the treating gastroenterologists when prescribing a systemic therapy in psoriasis patients with concomitant chronic inflammatory bowel disease (IBD).	**↑↑**	Strong consensus Consensus‐based
3.1‐2 | modified [2025] In patients with psoriasis and IBD, *we recommend* preferentially using approved targeted therapies with documented efficacy in these conditions: *Crohn's disease*: TNF inhibitors (infliximab, adalimumab, certolizumab^*^), anti‐IL12/23p40 (ustekinumab), or anti‐IL23p19 (risankizumab, guselkumab) *Ulcerative colitis*: TNF inhibitors (infliximab, adalimumab), anti‐IL12/23p40 (ustekinumab), or anti‐IL23p19 (risankizumab, guselkumab) ^*^At the time of updating the guideline to version 8, certolizumab pegol is not approved for the indication of Crohn's disease in Germany.	**↑↑**	Strong consensus^1^ Consensus‐based
3.1‐3 | modified [2025] If the agents of recommendation 3.1‐2 cannot be used, *we suggest* the following treatment options to be considered in patients with psoriasis and IBD: *Crohn's disease*: tildrakizumab *Ulcerative colitis*: tildrakizumab	**↑**	Strong consensus^1^ Consensus‐based
3.1‐4 | modified [2025] If the agents of recommendation 3.1‐2 cannot be used, *we suggest* the following oral treatment options to be considered in patients with psoriasis and IBD: *Crohn's disease*: methotrexate *Active ulcerative colitis*: ciclosporin (preferred), apremilast (also possible)	**↑**	Strong consensus^1^ Consensus‐based
3.1‐5 | reviewed [2025] In combination with other treatments, *we suggest* acitretin as an adjunct therapy for patients with IBD and psoriasis, especially in cases with mild paradoxical psoriasis.	**↑**	Strong consensus^1^ consensus‐based
3.1‐6 | reviewed [2025] *We suggest against* the use of IL17 inhibitors in patients with inflammatory bowel disease.	**↓**	Strong consensus^1^ Consensus‐based

^1^ Six abstentions due to conflicts of interest.

## CANCER: HOW SHOULD PSORIASIS PATIENTS WITH A HISTORY OF MALIGNANCIES BE MANAGED?

For the explanatory background text, see the long version.

**Table:** 

3.2‐1 | new [2025] *Cancer or remission < 5 years* *We recommend* discussing the decision to initiate immunosuppressive/ immunomodulatory therapies in psoriasis patients with a current diagnosis of cancer or a diagnosis made in the previous five years on a case‐by‐case basis with a physician specialized on the cancer entity and to reach a shared informed decision with the patients, respecting the patients’ preference. (In tumor entities with the risk of later metastasis and ongoing therapy, if applicable (for example, prostate), this applies also beyond the period of 5 years.)	**↑↑**	Strong consensus Consensus‐based
3.2‐2 | new [2025] *Remission > 5 years* For patients in remission of cancer for more than 5 years, *we recommend* selecting the most appropriate option for psoriasis treatment independent of the cancer. (For exceptions, see recommendations for “Cancer or remission < 5 years”)	**↑↑**	Strong consensus Consensus‐based
3.2‐3 | new [2025] *Palliative situation* For patients in palliative situation, *we recommend* treatment maximally maintaining the quality of life in coordination with the attending oncologist/ palliative care physician.	**↑↑**	Strong consensus Consensus‐based

*For the chapters “Depression”, “Kidney disease”, “Neurological diseases”, see the long version*.

## DIABETES: HOW SHOULD PSORIASIS PATIENTS WITH DIABETES MELLITUS BE MANAGED?

For the explanatory background text, see the long version.

**Table:** 

3.4‐1 | new [2025] If alternative treatments are available, *we suggest* prescribing alternatives to MTX in patients with type 2 diabetes (with concomitant metabolic syndrome and/or signs of liver damage).	**↑**	Strong consensus^1^ Consensus‐based
3.4‐2 | new [2025] If alternative treatments are available, *we suggest* prescribing alternatives to ciclosporin in patients with type 2 diabetes (with concomitant metabolic syndrome and/or signs of liver damage).	**↑**	Strong consensus^1^ Consensus‐based
3.4‐3 | reviewed [2025] *We suggest against* using acitretin or ciclosporin as first‐line treatment in patients with dyslipidemia.	**↓**	Strong consensus^1^ Consensus‐based

^1^ Three abstentions due to conflicts of interest.

## VIRAL HEPATITIS: WHEN AND HOW SHOULD PSORIASIS PATIENTS BE SCREENED FOR VIRAL HEPATITIS AND HOW SHOULD PATIENTS WHO TEST POSITIVE BE MANAGED?

A systematic review on the treatment of psoriasis patients with viral hepatitis was conducted. For details and a narrative synthesis of the identified evidence, we refer to Chapter 3 of the Evidence Report .

In addition, an alignment with and partial adaptation of the S3 guideline of the German Society for Gastroenterology, Digestive and Metabolic Diseases (DGVS) on prophylaxis, diagnosis and treatment of hepatitis B virus infection was conducted.^1^

The chapter was developed in cooperation with Prof. Dr. Markus Cornberg, Hannover Medical School, who was nominated by the German Society for Gastroenterology, Digestive and Metabolic Diseases (DGVS).

**Recommendations**:

**a) Screening**

**Table:** 

3.8‐1 | modified [2025] *We recommend against* screening for hepatitis A, D, or E as a routine measure before starting a systemic treatment.	**↓↓**	Strong consensus Consensus‐based Developed in cooperation with the DGVS

Screening for hepatitis A, D, and E should only be performed if this is indicated by medical history, elevated levels of liver enzymes, clinical signs and symptoms, but not as routine screening parameters.

**Table:** 

3.8‐2 | modified [2025] *We recommend* screening patients for hepatitis B (HBsAg, anti‐HBs, anti‐HBc) as a routine measure before starting a treatment with ciclosporin, deucravacitinib, methotrexate, or biologics.	**↑↑**	consensus Consensus‐based Developed in cooperation with the DGVS
3.8‐3 | new [2025] For the constellation anti‐HBc positive and HBsAg negative, *we recommend* excluding occult HBV infection by determination of HBV DNA.	**↑↑**	Strong consensus Consensus‐based Developed in cooperation with the DGVS
3.8‐4 | modified [2025] *We recommend* following the algorithm presented in Figure 1 for interpretation of hepatitis B test results.	**↑↑**	Strong consensus Consensus‐based Developed in cooperation with the DGVS
3.8‐5 | modified [2025] *We suggest* screening patients for hepatitis C as a routine measure before starting a treatment with deucravacitinib, methotrexate, or biologics.	**↑**	Strong consensus^1^ Consensus‐based Developed in cooperation with the DGVS
3.8‐6 | modified [2025] In case of positive findings for hepatitis C antibodies, *we recommend* testing for hepatitis C RNA.	**↑↑**	Strong consensus Consensus‐based Developed in cooperation with the DGVS
3.8‐7 | modified [2025] In case of positive findings for hepatitis C antibodies and hepatitis C RNA, *we recommend* referral to a specialist with expertise in treating viral hepatitis.	**↑↑**	Strong consensus Consensus‐based Developed in cooperation with the DGVS

^1^ Five abstentions due to conflicts of interest.

**FIGURE 1:**
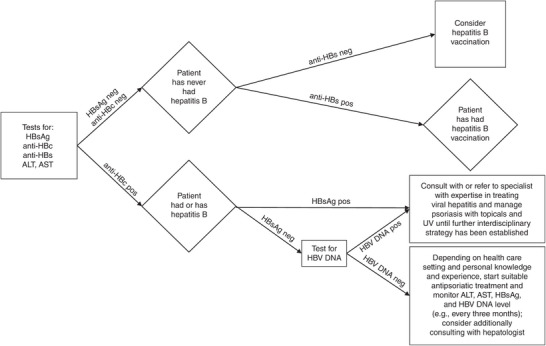
Algorithm for the interpretation of hepatitis B test results.

If viral hepatitis has been excluded once before initiating an immunosuppressive therapy and there is no history‐related/laboratory evidence of infection, re‐testing is not required when changing the therapy.

If, after a recent HCV treatment, a positive result for HCV antibodies and a negative test for HCV RNA are obtained, co‐assessment by a hepatologist/expert for liver diseases should be performed with respect to liver fibrosis.

A positive result for anti‐HCV antibodies and a negative test for HCV RNA without prior anti‐HCV treatment are evidence of a cured HCV infection. This will not require referral to a specialist with expertise in treating viral hepatitis.

**b) Treatment**

**Table:** 

3.8‐8 | modified [2025] ***H** **BsAg positive or HBV DNA positive*** *We recommend* that the treatment decision for patients with positive test result for HBsAg or positive HBV DNA is selected together with a specialist with expertise in treating viral hepatitis.* (*For indication of antiviral therapy with nucleos(t)ide analogs for prophylaxis of reactivation during immunosuppressive therapy, see: S3 guideline of the German Society for Gastroenterology, Digestive and Metabolic Diseases (DGVS) on prophylaxis, diagnosis and treatment of hepatitis B virus infection.)^1^	**↑↑**	Strong consensus Consensus‐based Developed in cooperation with the DGVS
3.8‐9 | new [2025] ***anti‐HBc positive, HBV DNA negative, and HBsAg negative*** ***anti‐HCV positive and HCV RNA negative*** The identified evidence does not allow for a specific recommendation of one of the psoriasis therapies discussed in the guideline in patients with the following serum status (hepatitis B: anti‐HBc positive/ HBsAg negative/ HBV DNA negative, or hepatitis C: anti‐HCV positive/ HCV RNA negative). For these patients, *we suggest* selecting the treatment option from the guideline most appropriate for the psoriasis disease of the patients while taking into account that the data on reactivation risk is still very limited for the newer drugs.	**↑**	Strong consensus^1^ Evidence‐ and consensus‐based (see Evidence Report, chapter 3) Developed in cooperation with the DGVS LoE: 3 (OECBM)

^1^ Five abstentions due to conflicts of interest.

The data identified during the systematic evidence search for this guideline is insufficient to give recommendations for or against using the available antipsoriatic drugs in patients with moderate‐to‐severe psoriasis and concomitant hepatitis B.

Table 13 in the Evidence Report provides a summary of the reported cases of reactivation. Reported cases need to be seen in correlation to approval date, especially with years and number of psoriasis patients with viral hepatitis exposed to the respective drug. This currently applies, in particular, to deucravacitinib where the number of exposed patients is still small. For detailed information, we refer to Guideline Development Report and Evidence Report. In the S3 guideline of the German Society for Gastroenterology, Digestive and Metabolic Diseases (DGVS) on prophylaxis, diagnosis and treatment of hepatitis B virus infection, the risk of HBV reactivation in patients with the status HBsAg negative/ anti‐HBc positive treated with MTX and TNFi is assessed as “low” and in those treated with ustekinumab as “moderate”. Other treatment options have not been assessed in this guideline. According to this guideline, these patients should be “tightly monitored in case of immunosuppression with moderate to low risk of reactivation” (see below), while “prophylactic antiviral therapy may be performed in special cases, for example, very long‐term immunosuppression, insufficient adherence to tight monitoring, or unfavorable risk factors (age, tumor entity, concomitant liver disease, or similar factors)”.^1^

For some treatment options, viral hepatitis is mentioned as contraindication in the summary of product characteristics (SmPC), although clinical practice, available case series, or registry data indicate a safety profile comparable to treatments where this is not mentioned as a contraindication. This holds true in particular for methotrexate, where study data do not indicate any increase in liver fibrosis.^2^

**c) Monitoring for reactivation during treatment**

**Table:** 

3.8‐10 | new [2025] For monitoring of reactivation of viral hepatitis in patients that are anti‐HBc positive/ HBsAg negative, *we recommend* regular testing for ALT, AST, HBsAg, and HBV DNA (at least every three months).	**↑↑**	Strong consensus Consensus‐based Guideline adaptation DGVS
3.8‐11 | reviewed [2025] *We recommend* recording all treatment initiations and follow‐up visits of psoriasis patients with concomitant hepatitis B or C cases in drug registries.	**↑↑**	Strong consensus Consensus‐based Developed in cooperation with the DGVS

Recommendation 3.8‐10 is an adaptation of a statement in the background text of the S3 guideline on prophylaxis, diagnosis and treatment of hepatitis B virus infection of the German Society for Gastroenterology, Digestive and Metabolic Diseases (DGVS)^1^ (AWMF registry number 021‐011).

Although the statement on the interval between follow‐up examinations was adapted based on the usual intervals between physician consultations in the addressed patient groups, it explicitly permits shorter follow‐up intervals.

## TUBERCULOSIS: HOW TO SCREEN FOR TUBERCULOSIS BEFORE AND DURING SYSTEMIC THERAPY?

This chapter is based on the previous versions of the guideline.^3^, ^4^, ^5^, ^6^ Details of the search are documented in Chapter 4 of the Evidence Report.

The chapter has been revised completely in cooperation with the German Central Committee against Tuberculosis (DZK) and the German Society for Pneumology and Respiratory Medicine (DGP).

Undetected and untreated, tuberculosis (TB) may present a life‐threatening disease. While TB is one of the most common infectious diseases worldwide with more than 10 million new cases per year, the disease is rare in Germany with 4,000 to 5,000 new cases per year.^7^, ^8^

However, the number of individuals with latent TB infections worldwide and in Germany can only be estimated.^9^ Only 5 % to 10 % of all infected people develop the disease during their lifetime. This risk is increased by diseases of the immune system and immunosuppressive drugs.

After the introduction of TNF inhibitors (TNFi), TB screening, which was not yet mandatory at the time, led to an increase in the incidence of complex and severe tuberculosis cases. This led to the introduction of screening before starting these therapies. For all immunomodulatory therapies developed subsequently, individuals with latent TB infections were excluded from pivotal studies or received preventive antituberculous treatment.^10^

Accordingly, assessments of the risk of reactivation are primarily based on mechanisms of action, the absence of reported cases of tuberculosis in patients receiving immunomodulatory therapies, international assessments,^11^, ^12^ and risk‐benefit analyses. Awareness of the potential symptoms of tuberculosis is still required in order to recognize, treat, and report any emerging cases of tuberculosis in a timely manner.

This chapter will focus on the screening for latent TB infection and the next chapter on the further course of action in case of a positive interferon‐gamma release assay (IGRA).

**Table:** 

3.9‐1 | new [2025] *We recommend* excluding latent TB infection using interferon‐gamma release assay (IGRA) before initiating treatment with a biologic agent or deucravacitinib**. **Deucravacitinib: due to lack of data concerning the reactivation risk	**↑↑**	Strong consensus^1^ Consensus‐based Developed in cooperation with the DGP
3.9‐2 | new [2025] In case of a negative IGRA, *we recommend* performing an additional chest X‐ray in order to exclude tuberculosis only, if: ‐ there is an increased probability for the presence of tuberculosis (see background text), or ‐ factors exist increasing the risk of a false negative IGRA test (see background text).	**↑↑**	Strong consensus Consensus‐based Developed in cooperation with the DGP

^1^ Three abstentions due to conflicts of interest.

*Factors increasing the risk of a false negative IGRA*:

‐ Immunosuppression (iatrogenic and/or disease‐related),^13^
‐ Lymphocytopenia,^14^
‐ Prior vaccination with live vaccine within the last 4–6 weeks,^15^
‐ Massive TB, for example, miliary tuberculosis.^16^

Until version 2021 of the German psoriasis guideline^6^, screening for latent TB infection was also recommended before initiating MTX therapy. This was not primarily due to concerns about reactivation of a latent TB infection during MTX treatment. It was intended as a precautionary measure for situations in which a switch from MTX to a biologic agent during the disease course was considered likely. This was based on the consideration that the test accuracy of an IGRA during MTX therapy might be reduced. This aspect may still be considered.

**Table:** 

3.9‐3 | new [2025] *We recommend against* repeating IGRA just based on fixed time intervals or due to switching from one drug to another without relevant risk of exposure.	**↓↓**	Strong consensus Consensus‐based Developed in cooperation with the DGP
3.9‐4 | new [2025] *We recommend* repeating IGRA during treatment if there is an increased risk of TB exposure (see background text: Indications for an increased probability of an exposure risk).	**↑↑**	Strong consensus Consensus‐based Developed in cooperation with the DGP

*Indications for an increased probability of TB exposure* ^15^ */presence of TB*:

‐ Direct contact with a person with active TB,
‐ Origin or longer stay (> 3 months) in a high‐prevalence country for tuberculosis (> 100 new cases/100,000 inhabitants),
‐ Stay or activities in an accommodation for migrants, asylum seekers, homeless people, or in enforcement,
‐ Working in a medical facility for TB patients,
‐ For further information, the guideline group refers to the S2k guideline “Tuberculosis in adulthood”^17^ (AWMF registry number: 020‐019) and to the information of the Robert Koch Institute (RKI; https://www.rki.de/DE/Content/InfAZ/T/Tuberkulose/Tuberkulose.html) and the German Central Committee against Tuberculosis (DZK; https://www.dzk‐tuberkulose.de/).

## TUBERCULOSIS: HOW SHOULD PSORIASIS PATIENTS WITH POSITIVE INTERFERON‐GAMMA RELEASE ASSAY (IGRA) BE MANAGED?

This chapter is based on the previous versions of the guideline.^3^, ^4^, ^5^, ^6^ Details of the search are documented in Chapter 4 of the Evidence Report.

This chapter has been revised completely in cooperation with the German Central Committee against Tuberculosis (DZK) and the German Society for Pneumology and Respiratory Medicine (DGP).

### Procedure in case of a positive finding in IGRA

**Table:** 

3.10‐1 | new [2025] In case of a positive IGRA, *we recommend* the following procedure to distinguish between active TB and latent TB infection: ‐ medical history: exposure risk, signs and symptoms of active TB (for example, cough, hemoptysis, fever, weight loss, night sweat), ‐ physical examination (for example, palpation of lymph nodes), ‐ chest X‐ray p.a., in two planes, if necessary.	**↑↑**	Strong consensus Consensus‐based Developed in cooperation with the DGP
3.10‐2 | new [2025] If there are abnormal clinical or unclear X‐ray findings or in case of radiographic indications of TB, *we recommend* referral to a specialist with expertise in treating tuberculosis.	**↑↑**	Strong consensus Consensus‐based Developed in cooperation with the DGP

*For diagnosis of tuberculosis, the guideline group refers to the S2k guideline “Tuberculosis in adulthood”* *17* *(AWMF registry number: 020‐019)*.

### Preliminary note on therapy selection

In case of existing tuberculosis, collaboration with pneumologists/infectiologists is important, for example, to discuss the requirement of interrupting or switching the antipsoriatic therapy.^17^

The following chapter will address the procedure for the further management of psoriasis vulgaris in case of latent TB infection and a potential preventive therapy. The recommendations will also consider the harmful and beneficial effects of preventive antituberculous therapy. Potential adverse drug reactions are listed in the S2k guideline “Tuberculosis in adulthood”^17^ (AWMF registry number: 020‐019).

### Selecting the suitable antipsoriatic therapy and indications for preventive antituberculous therapy

**Table:** 

3.10‐3 | modified [2025]For patients with latent TB infection, *we recommend* choosing one of the following options: ‐ acitretin, apremilast, ciclosporin, dimethyl fumarate, MTX, or phototherapy.	**↑↑**	consensus ^1^ Consensus‐based Developed in cooperation with the DGP
3.10‐4 | new [2025] *We suggest against* preventive antituberculous therapy** when initiating treatment with acitretin, apremilast, ciclosporin, dimethyl fumarate, or MTX. **If there are no other indications for initiating preventive antituberculous therapy (see S2k guideline “Tuberculosis in adulthood”^17^)	**↓**	consensus ^2^ Consensus‐based Developed in cooperation with the DGP

^1^ One abstention due to conflicts of interest

^2^ two abstentions due to conflicts of interest.

A documented search for systematic reviews (SRs) in the Medline database using Ovid identified no high‐quality SRs on the risk of reactivation when using acitretin, ciclosporin, fumarates, or MTX.

These drugs have been used for decades (exception: apremilast since 2015). To date, neither screening for latent TB infection nor preventive antituberculous therapy has been recommended for the drugs acitretin, apremilast, ciclosporin, and dimethyl fumarate in the German psoriasis guideline.^6^ In addition, screening for latent TB infection or tuberculosis is not recommended in the SmPCs. Data from health services research indicate that this practice is followed in the majority of cases.^18^ The guideline group has no knowledge of any safety signals resulting from this approach, whether from clinical experience or from the available literature.

In contrast, for MTX, data from health services research showed considerable variation in the use of preventive antituberculous therapy in cases of latent TB infection.^18^

Since the 1950s, MTX has been used for numerous hemato‐oncological, rheumatological, immunological, and dermatological diseases. The SmPC for MTX did not generally require explicit tuberculosis screening or preventive antituberculous therapy.

It can therefore be assumed that a large number of patients worldwide with latent TB infection have been treated with this drug. The guideline group is not aware of any increase in the number of cases with severe or atypical disease course, as reported during TNFi treatment.^19^

No recent systematic review of sufficient quality on assessment of the reactivation risk of latent TB infection during MTX treatment could be found. Two population‐based case‐control studies were identified by manual search.^20^, ^21^ In one of these, the reactivation risk in patients with rheumatoid arthritis treated with MTX was estimated to be low.^21^ In the other, no association between MTX and an increased risk of active tuberculosis was found in patients with rheumatoid arthritis ≥ 67 years of age.^20^

The evidence identified is considered insufficient to identify a relevant risk of reactivation or a risk of particularly severe or atypical disease courses and to justify a recommendation for preventive antituberculous therapy.

Factors arguing against preventive antituberculous therapy in latent TB infection during MTX treatment include potential harm due to the preventive antituberculous therapy, potential drug interactions, and the considerable effort associated with the intake for several months.

Considering the aspects mentioned above and taking its clinical experience into account, the guideline group decided to recommend against preventive antituberculous therapy for the agents mentioned in recommendation 3.10‐4, unless indications mentioned below exist (see section: “General indication for preventive antituberculous therapy in patients with latent TB infection”).

**Table:** 

3.10‐5 | modified [2025] For patients with latent TB infection, *we suggest* choosing one of the following options: IL17i, IL23i, or IL12/23p40i.	**↑**	Strong consensus^1^ Evidence‐ and consensus‐based (see Evidence Report, chapter 4) LoE: 3‐4 (OCEBM) Developed in cooperation with the DGP
3.10‐6 | new [2025] *We suggest* preventive antituberculous therapy when initiating a therapy with IL12/23p40i**. **Deviation from SmPC; according to SmPC, preventive antituberculous therapy “must” be initiated for ustekinumab. The guideline group could not identify any data sufficiently justifying this differentiation. Informing the patient and including this aspect in the shared decision‐making are required.	**↑**	Strong consensus^1^ Consensus‐based Developed in cooperation with the DGP
3.10‐7 | new [2025] A preventive antituberculous therapy may be considered when initiating a therapy with IL17i or IL23i.	**0**	Strong consensus^1^ Consensus‐based Developed in cooperation with the DGP

^1^ Five abstentions due to conflicts of interest.

Generating data on the reactivation risk of newer agents is only possible to a limited extent or not at all, given that patients with latent TB infection were already excluded or received preventive antituberculous treatment in pivotal trials (see chapter on TB screening).

Recommendation 3.10‐5 is based on a systematic review (SR), identified in a systematic search, that examined the reactivation risk of latent TB infection in patients with psoriasis and therapy with biologics with and without preventive therapy.^22^

Given that the authors of the SR^22^ did not differentiate in their evidence synthesis whether the reactivations occurred on preventive antituberculous therapy or not, the guideline group decided to re‐extract this information from the primary studies included in the SR (for details, see Evidence Report, Chapter 4).

In the view of the guideline group, this analysis provided insufficient evidence for a reactivation risk with IL17 or IL23 inhibitors. For this class of drugs, the SmPC considers initiation of preventive antituberculous therapy as optional. Other international experts also estimate the reactivation risk of this class of drugs to be lower.^11^, ^12^

When initiating treatment with the IL12/23p40 inhibitor ustekinumab, the SmPC requires preventive antituberculous therapy in case of latent TB infection. Upon inquiry, the manufacturer was unable to provide any data with specific evidence for an increased risk of reactivation. In the identified data,^22^ the guideline group detects no evidence for an increased risk of reactivation. However, the examined case numbers were very low (see Evidence Report, Chapter 4).

**Table:** 

3.10‐8 | new [2025] *We suggest against* the use of deucravacitinib** in patients with latent TB infections unless there are no other suitable treatment options. **Due to lack of data concerning the reactivation risk	**↓**	Strong consensus^1^ Consensus‐based Developed in cooperation with the DGP
3.10‐9 | new [2025] *We suggest* preventive antituberculous therapy when initiating a therapy with deucravacitinib**. **Due to lack of data concerning the reactivation risk	**↑**	consensus ^1^ Consensus‐based Developed in cooperation with the DGP

^1^ Two abstentions due to conflicts of interest.

The data on the reactivation risk of a latent TB infection during therapy with deucravacitinib are still insufficient for making an assessment. The SmPC recommends initiating preventive antituberculous therapy. Due to the absence of other experience and the unknown reactivation risk, the guideline group concurs with this recommendation.

**Table:** 

3.10‐10 | reviewed [2025] *We recommend against* the use of TNFi in patients with latent TB infection unless there are no other suitable treatment options.	**↓↓**	Strong consensus^1^ Consensus‐based Reviewed in cooperation with the DGP
3.10‐11 | new [2025] *We recommend* preventive antituberculous therapy when initiating a therapy with TNFi.	**↑↑**	consensus ^1^ Consensus‐based Developed in cooperation with the DGP

^1^ Two abstentions due to conflicts of interest.

A systematic review identified in manual search shows that the risk of active tuberculosis is increased with TNFi compared to the control group (non‐TNFi or placebo) (46/7,912 vs 3/3,967; OR 1.94 (95 % confidence interval [CI] 1.1; 3.44), I^2^ = 0 %, N = 29 RCTs)^23^. An increased incidence of extrapulmonary and disseminated manifestations with TNFi has been reported.^19^

### General indication for preventive antituberculous therapy in patients with latent TB infection

For verification of the indication for preventive antituberculous therapy in case of latent TB infection – also independent from initiation of a psoriasis therapy – the guideline group refers to the S2k guideline “Tuberculosis in adulthood”^17^ (AWMF registry number: 020‐019).

Indications for preventive antituberculous therapy of latent TB infection exist in particular for (adapted from Schaberg et al.^17^):

‐ contact persons of individuals with active TB,
‐ people with insufficient control of HIV disease,
‐ people prior to TNFi therapy and potentially other biologics/JAK inhibitors,
‐ people with severe underlying diseases requiring immunosuppression,
‐ people prior to or after a scheduled solid organ hematological transplantation,
‐ people from countries with a high prevalence of TB.

### Form of preventive antituberculous therapy

**Table:** 

3.10‐12 | new [2025] *We recommend* administration of either rifampicin (4 months) or isoniazid + rifampicin (3 months) or isoniazid (9 months) as preventive antituberculous therapy.	**↑↑**	Strong consensus Consensus‐based Developed in cooperation with the DGP

The recommendations concerning type and duration of preventive antituberculous therapy are based on the S2k guideline “Tuberculosis in adulthood”^17^ (AWMF registry number: 020‐019). In addition, the guideline group refers to this guideline for further interventions that need to be considered before, during, and after the preventive antituberculous therapy. The shorter regimens of rifampicin‐containing therapies are usually preferred given that it is easier to ensure adherence. Interactions and, if necessary, therapy adjustments of other medications must be considered when using rifampicin.

**Table:** 

3.10‐13 | new [2025] *We suggest* to generally observe an interval of 4 weeks between start of preventive antituberculous therapy and initiation of the immunosuppressive therapy.	**↑**	Strong consensus Consensus‐based Developed in cooperation with the DGP

The interval of 4 weeks generally recommended between initiating preventive antituberculous therapy and immunosuppressive therapy is not based on study data, but on theoretical considerations. The guideline group deliberately issues a weakened recommendation and emphasizes that shorter intervals between starting preventive antituberculous therapy and initiating antipsoriatic systemic therapy are also considered feasible in cases of psoriasis requiring urgent treatment.

### Shared decision‐making

Initiation of a preventive antituberculous therapy requires a risk‐benefit assessment together with the patient (Table 4). This is presented in detail in the guideline on therapy of tuberculosis.^17^ The patient must be informed about the tuberculosis‐specific symptoms and the remaining risk of recurrent infection/disease even after performing preventive antituberculous therapy.

**TABLE 4:** Potential reasons for or against preventive antituberculous therapy.

Reasons for preventive antituberculous therapy	Reasons against preventive antituberculous therapy
Younger age, general prevention of reactivation in case of enhanced life‐time risk of tuberculosis (see S2k guideline “Tuberculosis in adulthood”^17^)	Increased risk of hepatotoxicity (for example, age > 60 years (especially relevant for therapy with INH), known liver disease)
Additional immunosuppression	Extensive comedication, risk of cumulative SAEs, drug interactions
Planned therapy with higher risk of reactivation	Planned therapy with low risk of reactivation
Relevant contact to an index case with positive IGRA and negative findings in chest X‐ray	Adherence not guaranteed

Based on a network meta‐analysis^24^ and the statements of national and international guidelines,^17^, ^25^ the guideline group considers preventive antituberculous therapy to be effective, although the results will differ depending on therapy selection and duration.

### Promotion of health services research

**Table:** 

3.10‐14 | new [2025] *We recommend* enrolling patients with latent TB infection in registries/scientific studies after initiation of treatment with IL17i, IL23i, or IL12/23p40i.	**↑↑**	Strong consensus Consensus‐based Developed in cooperation with the DGP

### Management of active tuberculosis

**Table:** 

3.10‐15 | new [2025] In case of existing active TB, *we recommend* developing a therapeutic concept for psoriasis and TB in cooperation with a specialist with expertise in treatment of tuberculosis.	**↑↑**	Strong consensus Consensus‐based Developed in cooperation with the DGP

*For further information, the guideline group refers to the S2k guideline “Tuberculosis in adulthood”* *17* *(AWMF registry number: 020‐019)*.

*For the chapters “Wish for child/pregnancy”, “Psoriasis arthritis”, and “Immunogenicity: Development of antibodies against targeted therapies in psoriasis”, see the long version*.

## VACCINATIONS: HOW SHOULD VACCINATIONS BE MANAGED IN PSORIASIS PATIENTS ON SYSTEMIC TREATMENT?

A non‐systematic literature review was performed in February 2023.

**Results/recommendations**:

The guideline group considers a psoriasis disease not *per se* as reason to deviate from the standard vaccination recommendations/national vaccination policies.

For patients starting a systemic immunomodulatory therapy for psoriasis:

‐ The optimal time for vaccinations is before starting a systemic immunosuppressive therapy.
‐ Check need for vaccinations and perform respective vaccinations according to the national vaccination policies before starting a systemic immunosuppressive therapy, if possible.
‐ Check the specific SmPCs with respect to the recommended interval for starting a systemic immunosuppressive medication after vaccination.

For patients receiving a systemic immunosuppressive therapy for psoriasis:

‐ The immune response to vaccinations is affected by several factors including type and dose of the systemic immunosuppressive therapy, type of the vaccine (live or inactivated vaccine), intrinsic factors (for example, age, comorbidities), and extrinsic factors (for example, existing immunity due to previous antigen exposure).^26^
‐ Check national vaccination policies for vaccination requirements during therapy.
‐ Check SmPCs concerning the recommended time interval for administration of a systemic immunosuppressive therapy after vaccination.
‐ In general, inactivated vaccines can be used safely in patients receiving systemic immunosuppressive therapy. However, the immunogenicity of the vaccine may be reduced. Live vaccines should be avoided in accordance with the respective SmPCs. Live vaccines should also be avoided in infants (up to six months of age) whose mothers received biologic therapy after the 16th week of gestation (see specific SmPCs and chapter on pregnancy).

We recommend complete COVID‐19 vaccination including an additional (third) basic dose and booster vaccinations according to the national vaccination policies, given that patients receiving immunosuppressive therapies may have an attenuated humoral or cellular reaction to the COVID‐19 vaccine compared to healthy individuals.^27^, ^28^, ^29^ Withholding MTX therapy for 2 weeks after vaccination should be considered, if possible, given that this may improve the immunogenicity of the vaccine. However, the positive effect of this measure on the clinical effectiveness of vaccines has not been shown in studies.^30^, ^31^ There is no consensus on the question whether discontinuation of methotrexate will increase the protection against infection or reduce the probability of a symptomatic disease or a severe course of COVID‐19.

## Note on Guideline adaptation

The authors of this work have adapted, remixed, transformed, translated or built upon a previous version of the following article: EUROGUIDERM GUIDELINE FOR THE SYSTEMIC TREATMENT OF PSORIASIS by Nast A et al.; which is available in its final form on the European Dermatology Forum website (https://www.guidelines.edf.one/guidelines/psoriasis-guideline) (licensed under CC BY NC 4.0, https://creativecommons.org/licenses/by-nc/4.0/):

– A Nast, PI Spuls, C Dressler, Z Bata‐Csörgö, I Bogdanov, H Boonen, EMGJ De Jong, I Garcia‐Doval, P Gisondi, D Kaur‐Knudsen, S Mahil, T Mälkönen, JT Maul, S Mburu, L Mercieca, U Mrowietz, A Pennitz, E Remenyik, D Rigopoulos, PG Sator, M Schmitt‐Egenolf, M Sikora, K Strömer, O Sundnes, G Van Der Kraaij, N Yawalkar, C Zeyen, C Smith. EUROGUIDERM GUIDELINE FOR THE SYSTEMIC TREATMENT OF PSORIASIS VULGARIS September 2023, partial update February 2025.

Furthermore, this article is based on an adaptation of the previous version of the German version of the guideline, which has been published in its final form at https://doi.org/10.1111/ddg.14508 and https://doi.org/10.1111/ddg.14507:

– Nast A, Altenburg A, Augustin M, Boehncke WH, Härle P, Klaus J, Koza J, Mrowietz U, Ockenfels HM, Philipp S, Reich K, Rosenbach T, Schlaeger M, Schmid‐Ott G, Sebastian M, von Kiedrowski R, Weberschock T, Dressler C. German S3‐Guideline on the treatment of Psoriasis vulgaris, adapted from EuroGuiDerm – Part 1: Treatment goals and treatment recommendations. J Dtsch Dermatol Ges. 2021 Jun;19(6):934‐150. doi: 10.1111/ddg.14508.
– Nast A, Altenburg A, Augustin M, Boehncke WH, Härle P, Klaus J, Koza J, Mrowietz U, Ockenfels HM, Philipp S, Reich K, Rosenbach T, Schlaeger M, Schmid‐Ott G, Sebastian M, von Kiedrowski R, Weberschock T, Dressler C. German S3‐Guideline on the treatment of Psoriasis vulgaris, adapted from EuroGuiDerm – Part 2: Treatment monitoring and specific clinical or comorbid situations. J Dtsch Dermatol Ges. 2021 Jul;19(7):1092‐1115. doi: 10.1111/ddg.14507.

Please note: The present adapted guideline did not undergo an approval procedure by the European Dermatology Forum, but has been approved by the editing German societies. This guideline is subject to the provisions of Creative Commons Attribution – NonCommercial license.

## CONFLICT OF INTEREST STATEMENT

For the authors of the German Version: see Guideline Development Report of the German adaptation at www.awmf.org. For the authors of the EuroGuiDerm version: see Methods Report: https://www.guidelines.edf.one/guidelines/psoriasis‐guideline [accessed: April 10, 2025].
